# Impact of Adequate Disinfection Techniques for Ultrasound-Guided Injections in Musculoskeletal Rehabilitation: A Scoping Review

**DOI:** 10.3390/diagnostics15070933

**Published:** 2025-04-05

**Authors:** Angelo Alito, Alessandro de Sire, Marco Di Gesù, Enrico Buccheri, Daniele Borzelli, Rita Chiaramonte, Umile Giuseppe Longo, Antonio Ammendolia, Michele Vecchio, Daniele Bruschetta

**Affiliations:** 1 Department of Biomedical, Dental Sciences and Morphological and Functional Images, University of Messina, 98122 Messina, Italy; alitoa@unime.it (A.A.); daniele.borzelli@unime.it (D.B.); dbruschetta@unime.it (D.B.); 2 Physical and Rehabilitative Medicine, Department of Medical and Surgical Sciences, University of Catanzaro “Magna Graecia”, 88100 Catanzaro, Italy; ammendolia@unicz.it; 3 Research Center on Musculoskeletal Health, MusculoSkeletalHealth@UMG, University of Catanzaro “Magna Graecia”, 88100 Catanzaro, Italy; 4 Health Center Mya Salute, 90146 Palermo, Italy; digesumarco@gmail.com; 5 Department of Biomedical and Biotechnological Sciences, University of Catania, 95123 Catania, Italy; enrico.buccheri@gmail.com (E.B.); ritachiaramd@gmail.com (R.C.); michele.vecchio@unict.it (M.V.); 6 Fondazione Policlinico Universitario Campus Bio-Medico, Via Alvaro del Portillo 200, 00128 Roma, Italy; g.longo@policlinicocampus.it; 7 Research Unit of Orthopaedic and Trauma Surgery, Department of Medicine and Surgery, Università Campus Bio-Medico di Roma, Via Alvaro del Portillo 21, 00128 Roma, Italy; 8 Rehabilitation Unit, AOU Policlinico G. Rodolico-San Marco, 95123 Catania, Italy

**Keywords:** interventional physiatry, intraarticular injections, physical and rehabilitation medicine, sterility, ultrasound-guided procedures

## Abstract

**Background**: Interventional physiatry is a branch of medicine that uses minimally invasive ultrasound-guided techniques for diagnosis and treatment in the musculoskeletal system. The aim of this scoping review is to investigate the sterilisation techniques used and the rate of infection with ultrasound-guided injections. **Methods**: PubMed was searched up to 30 September 2024 using the following search terms (“Ultrasound, Interventional”[mesh]) AND “Injections, Intra-Articular”[mesh]; “Ultrasound-guided intra-articular injection”. The inclusion criteria were randomised clinical trials, written in English, involving US-guided mini-invaexercissive procedures. **Results**: The search identified a total of 256 potentially relevant publications. After screening for duplication, inclusion, and exclusion criteria, 105 articles were eligible for data extraction. In 51 studies, the method of skin disinfection was not specified, 18 RCT reported a ’sterile condition’, 9 studies used povidone–iodine solution, 5 used alcohol, and 2 used chlorhexidine 0.5%. In 64 trials, the method of probe preparation was not specified, 11 trials described the use of sterile gel, 10 trials reported the use of a probe cover, sterile pad, or barrier, and 2 trials reported the use of chlorhexidine 0.5%; 41 studies reported mild adverse events and 4 serious adverse events. **Conclusions**: Taken together, the findings of this scoping review did not show a clear relationship between current sterilisation protocols and the prevention of the microbial contamination of the probes or the patient’s skin. The variation in protocols highlights the need for standardised guidelines and more rigorous studies to accurately determine the most effective disinfection practices.

## 1. Introduction

Interventional physiatry is a subspecialty of physical and rehabilitation medicine (PRM) that uses minimally invasive techniques, predominantly ultrasound (US)-guided procedures, to diagnose and treat pain and musculoskeletal (MSK) disorders [1].

This rapidly evolving field integrates US into routine clinical practice, significantly enhancing the assessment and management of various MSK conditions [2,3].

US has become the preferred imaging modality for assessing soft tissue structures, evaluating disease progression, and improving the precision of therapeutic interventions due to its non-invasive characteristics, real-time imaging capabilities, and lack of radiation exposure [4].

US can be applied to any region of the body, allowing for precise probe placement and dynamic imaging evaluation for additional functional insight [5,6].

Its portability, safety, and lack of radiation make it suitable for patients of all ages, including newborns, elderly, pregnant women, and those with metal implants or pacemakers [7,8].

The direct, real-time visualisation of anatomical targets allows accurate invasive or minimally invasive interventions enhancing accuracy, reducing complications, and improving clinical outcomes.

The US provides a clear visualisation of the peripheral nerves and surrounding structures, facilitating the correct delivery of anesthetics or steroids. US-guided nerve blocks increase procedural success, reduce the required anesthetic dose, and shorten onset time [9].

Furthermore, the precision in targeting intra-articular spaces improve injection treatment (e.g., platelet-rich plasma (PRP), hyaluronic acid, and collagen) efficacy and outcomes in inflammatory and degenerative conditions [10,11,12,13], allowing the physiatrist to be more precise, avoiding potential complications related to vessel or nerve damage [3,14].

US guides needle placement during tendon interventions such as percutaneous tendon injections, galvanic electrolysis, percutaneous lavage for calcific rotator cuff tendinopathy, hydro-dissection for adhesions, and regenerative therapy, reducing the risk of tendon rupture by avoiding unintended intratendinous injections [15,16].

US is useful for guiding PRP or stem cell injections into injured muscle tissue to stimulate healing and regeneration [17,18].

US is particularly beneficial in local spasticity treatment with botulinum toxin (BoNT), helping to accurately target spastic muscles, even when anatomical landmarks are altered by conditions like stroke or cerebral palsy [19,20], ensuring precise needle placement, minimising risks to adjacent structures, and improving therapeutic outcomes [21].

Therefore, the most used interventional procedures are intra-articular or peri-tendinous injections of substances such as steroids, collagen, hyaluronic acid, and oxygen–ozone therapy [22,23]. The use of US guidance in the MSK field has grown in recent decades due to the improved efficacy, safety, and cost effectiveness compared to blind injections [24].

At the same time, given the infection risk associated with interventional procedures, such as an estimated incidence of 4.6 infections per 100,000 corticosteroid injections in the United States, the question has arisen on whether the use of US guidance might influence infection rates [25,26].

To reduce infection risk, the Spaulding classification system remains valid today and appears to indicate appropriate disinfection and sterilisation methods for medical devices used in safe interventional procedures [27].

Although general guidelines for invasive procedures exist, there is no standardised protocol specifically for US-guided injections and various sterilisation techniques have been proposed to minimise the risk of infection. The use of sterile gloves, the sterilisation of the skin with chlorhexidine or povidone–iodine, covering the probe with a sterile glove, and the use of sterile gel instead of normal gel are all options that theoretically make the operating field more sterile [28].

Other options include, in addition to normal skin sterilisation, using chlorhexidine, povidone–iodine, or both instead of US gel, and using ordinary kitchen cling film instead of a sterile glove when using chlorhexidine [29,30].

Although these procedures are often performed outside the operating room, maintaining rigorous sterility standards, proper probe cleaning, skin disinfection, and correct injection techniques are essential in order to avoid infection [29].

The impact of sterile techniques on US-guided injections remains a topic of concern due to inconsistent data and variability among studies. Balancing precision with aseptic practices is crucial in order to prevent serious infections such as septic arthritis, cellulitis, or systemic infections [31].

Sterilisation protocols can vary based on physician experience and procedure specifics, leading to inconsistencies between studies and real clinical settings.

The aim of this scoping review was to conduct a comprehensive literature review of randomised trials utilising US-guided injection techniques in the MSK and rehabilitation field. The objective was to evaluate the incidence of complications and infections, specifically in relation to the disinfection (or sterilisation) techniques employed.

## 2. Materials and Methods

### 2.1. Study Design

This scoping review was structured around the research question “What methods are used to ensure sterility in interventional physiatry?” and was conducted according to the standards of the Preferred Reporting Items for Systematic reviews and Meta-Analyses extension for Scoping Reviews (PRISMA-ScR) [32].

### 2.2. Search Strategy

A comprehensive literature search was conducted in PubMed up to 30 September 2024. The search strategy combined relevant terms associated with sterility in interventional physiatry, using the following search strings (“Ultrasonography, Interventional”[Mesh]) AND “Injections, Intra-Articular”[Mesh], as well as the phrase “ultrasound guided intra articular injection”.

### 2.3. Inclusion and Exclusion Criteria

The inclusion criteria were (a) randomised clinical trial (RCT), (b) in English, (c) involving US-guided mini-invasive procedures or requiring needles. Exclusion criteria included grey literature, review articles, animal studies, and articles not available as full text.

### 2.4. Selection of Sources of Evidence

**Figure 1:**
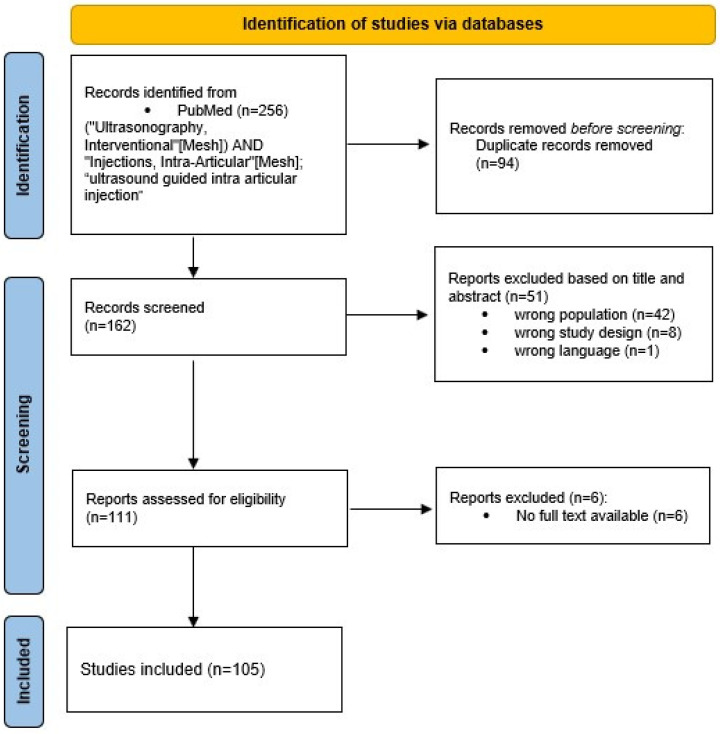
Flowchart summarising the selection process of papers, with the Preferred Reporting Items for Systematic reviews and Meta-Analyses extension for Scoping Reviews (PRISMA-ScR).

Two independent reviewers performed the literature search and article selection according to the eligibility criteria. Initially, identified publications were imported into Rayyan online software for the simultaneous evaluation of studies [33]. The software was then used to identify and remove duplicate articles. Titles and abstracts were then screened independently by the reviewers. Articles meeting the inclusion criteria were selected for full-text reading. Any disagreements between reviewers were resolved through consensus. A PRISMA flowchart summarising the selection process is provided in Figure 1.

### 2.5. Data Extraction

Data extraction was then independently completed by two authors, and data accuracy was verified by an additional author. Disagreements were resolved by consensus. Relevant data were then entered into a database with the agreement of the observers on (1) authors, year, and title, (2) sample size and characteristics, (3) intervention, (4) skin disinfection, (5) probe US disinfection, and (6) side effects.

### 2.6. Synthesis of Results

The extracted data were summarised according to the sterility methods used for skin and ultrasound probes, including the number of studies that did not report specific sterilisation methods.

## 3. Results

### 3.1. Study Selection

The search of the scientific literature in the PubMed database yielded a total of 256 potentially relevant publications, and 94 duplicates were excluded from the search prior to screening. After reading the title and abstract, 51 articles were discarded for the following reasons: wrong target population (*n =* 42), wrong study design (i.e., study protocol, letter to the editor, and review article) (*n =* 8), and wrong language (*n =* 1). Within the remaining 111 articles, a total of 6 studies were not retrieved. The remaining 105 articles were screened by reading the full text: all were eligible for data extraction, and all were included in this review (see Figure 1 for further details). A table with an overview of the included studies and a summary of their results is provided in Supplementary Materials.

### 3.2. Patients’ Characteristics

The total number of patients in the pooled sample of the 102 included RCTs was 7155. Osteoarthritis (OA) was the most representative disease, with 2989 patients affected, mainly knee OA (1151). The shoulder was the most treated site, with glenohumeral and acromion-clavicular OA (115), adhesive capsulitis (AC) (992), tendinopathy (637), pain syndromes (1483), subacromial-subdeltoid bursa (SASD) bursitis (186), subacromial impingement syndrome (281), and hemiplegic shoulder pain (111). Tendinopathies were reported in 1001 patients, mainly for the rotator cuff (606) and tennis elbow (203). Carpal tunnel syndrome was reported in 232 patients. An overview is provided in Table 1.

### 3.3. Intervention

The procedures performed under US guidance were 76 intra-articular injections, 16 peri-tendinous injections, 5 intra-bursal injections, 4 perinervous injections, 2 dry needling, and 1 arthrocentesis.

### 3.4. Skin Preparation, Disinfection, and Sterility

In 52 trials, the method of skin disinfection was not specified, 19 RCT reported a generical ‘sterile condition’, 9 trials used povidone–iodine solution, 5 used alcohol, and 2 used chlorhexidine 0.5%. Other methods were described, such as the ‘no touch technique’ (2) or ‘aseptic technique’ (5); and 2 papers described the use of ChloraPrep^®^, a sterile antiseptic solution consisting of a mixture of 2% chlorhexidine gluconate in 70% isopropyl alcohol. Only 3 studies described the use of drapes to create sterile fields.

### 3.5. US Probe Preparation

In 66 studies, the method of the probe setup was not specified; 11 papers described the use of gel, but only 9 specified that it was sterile gel; 10 studies reported the use of a probe cover, sterile pad, or barrier; 2 studies reported the use of chlorhexidine 0.5% to disinfect the probe; and 4 reported the use of sterile gloves.

### 3.6. Adverse Effects

Overall, 43 trials reported no major or minor side effects. Of the trials reporting mild adverse events, the most common were pain (10), flushing (7), skin atrophy/changes (6), mild vagal reactions (6), and nausea (3); and 32 trials did not report whether side effects occurred. In the Paskins et al. study, one event was considered to be possibly related to the study treatment: a participant with a bioprosthetic aortic valve died of subacute bacterial endocarditis four months after treatment [34]. In the Paget study, one transient ischemic attack was reported in the placebo group, which was not related to the injection [35]. Roddy and colleagues reported in their study that one participant who received a US-guided injection was hospitalised with pyelonephritis [36]. In the Atchia study, there was one case of femoral head collapse in a patient who did not receive an injection and one confirmed case of post-arthroplasty infection [37]. In the paper by Galiano et al., one patient developed fluid retention with oedema of the legs and arms, but it is unclear whether these symptoms were due to a side effect of steroids [38]. Jurgensmeier reported gastrointestinal bleeding after a patient increased his warfarin dose about 2 months after ketorolac injection [39].

## 4. Discussion

Interventional physiatry is a relatively new subspecialty of PRM that has been growing in recent years and typically uses US-assisted procedures such as intra-articular or peri-tendinous infiltrations [40,41]. US has been established as an essential tool in PRM, significantly improving the evaluation, treatment, and interventional management of MSK disorders. Its ability to offer real-time, high-resolution images increases diagnostic and procedural accuracy, ultimately leading to better patient outcomes [4]. The aim of this paper was to analyse the current literature on RCTs of US-guided interventional procedures, to highlight the most used sterility protocols and the related risk of infections.

Therapeutic intra-articular injections are generally well-tolerated, safe, and effective, but iatrogenic infections are possible [42,43]. Therefore, there is a paucity of data to support a sterilisation method that minimises potential complications while maintaining therapeutic efficacy [28].

The widespread use of US enables physiatrists to improve both diagnosis and treatment in the field of PMR, with several advantages in the physiatrist’s assessment.

The rapid and real-time visualisation of soft tissues, muscles, tendons, ligaments, nerves, and joints, which can aid diagnosis, helps to identify the extent and precise location of injuries [3].

US allows for a dynamic assessment, observing structures in motion or conflicts between structures, or joint stability and muscle function. In addition, it is a non-invasive, cost-effective, accessible, and safe diagnostic tool because it uses sound waves rather than ionising radiation, making it an easily reproducible technique, even in pregnant women or children [44].

US can help and guide the physician in the better diagnosis and planning of rehabilitation treatment, following the evolution of different pathologies, improving patient engagement and adherence to rehabilitation protocols [45].

Finally, one of the major US advantages in rehabilitation medicine is its use in guiding interventions. Procedures such as injections (e.g., corticosteroids and hyaluronic acid), aspirations, and nerve blocks can be performed with greater accuracy when US guidance is used [46]. This not only increases the effectiveness of the treatment, but also reduces the risk of complications by ensuring the precise delivery of drugs or other therapeutic agents [3]. In addition, in procedures involving BoNT, the efficacy of US can be further enhanced by a thorough anatomical knowledge of the intramuscular nerve distribution. Yi et al. demonstrated that nerve arborisation in the latissimus dorsi is highly localised, highlighting the importance of targeting neuromuscular junction-rich zones [47].

To prevent contamination and microbial infiltration during US-guided procedures, ensuring proper sterility protocols is essential. Local probe disinfection helps maintain hygiene and avoid cross-contamination, but improper techniques or inappropriate disinfectants can result in probe damage, impairing its functionality and longevity [48,49].

Improper disinfection techniques may damage the US probe surface by causing cracks or leaving sticky residues, potentially increasing the risk of infection due to surface contamination [50]. Other damage could affect the acoustic lens, reducing sensitivity and possibly distorting the US signal, resulting in a reduced image quality and less accurate diagnostic capability [51]. In addition, using proper cleaning techniques, such as wiping gently with appropriate solutions and avoiding excessive pressure, can help maintain the integrity of the probe.

On this basis, and given that there is no clear superiority of a sterility protocol that includes the US probe, it might be interesting to propose a sterility protocol that also takes into account cost/effectiveness aspects, such as the use of a non-ferrous patch as a sterile probe cover proposed by Deshmukh et al. [30], plus the use of chlorhexidine on the skin as an alternative to the more expensive protocol of a sterile probe cover patch and sterile gel [29].

While there are well-established sterility protocols for percutaneous and surgical procedures, guidelines for US-assisted procedures remain inconsistent. Some recommend the use of chlorhexidine or iodopovidone, while others endorse the use of protective films, sterile probe covers, or sterile gel [52,53,54,55].

The choice between sterile and non-sterile US gel remains controversial. While some studies suggest that sterile gel may reduce the risk of contamination, others report increased bacterial growth associated with its use. Sherman et al. [26] found that sterile US gel resulted in greater skin contamination compared to non-sterile gel, whereas Provenzano et al. [56] reported a low baseline contamination rate with non-sterile gel. Given these conflicting results, also considering the previous evidence on this topic [57,58,59,60,61], further research is needed to establish clear guidelines for gel selection to minimise the risk of infection during ultrasound-guided procedures.

The results of this scoping review highlight significant inconsistencies in the reporting and implementation of sterility protocols for US-guided interventional procedures. Where sterility practices were detailed, they varied widely and this variation highlights the lack of a universal standard, making it difficult to compare results and assess best practices across different clinical settings. Moreover, there were no specific data on the prescription or suggestion of physical exercise after the injections, despite the well-known role both in athletes of patients with osteoarthritis [62,63,64,65,66,67].

To date, few studies compare different sterilisation protocols in large patient cohorts, and the available research lacks consensus on the superiority of any specific method.

About half of the studies included (54/105) do not report skin sterility conditions and almost as many (41/105) omitted details of probe sterilisation. Even when sterility protocols were mentioned, they were often described in vague terms such as “sterile conditions” without further specification. Adverse events were also poorly reported (32 articles do not report them and 41 report, generically, “no adverse events”). Serious infections or complications appeared to be rare, with only seven cases reported out of 6908 patients treated, regardless of the type of procedure or sterility protocol used. Moreover, differences in how infection rates are reported and variations among patient populations make interpreting the results challenging.

While infection rates remain low across studies, the variability in sterility protocols poses the question of how to tailor disinfection to procedural risk. Low-risk procedures, such as peri-tendinous injections, may require more basic measures, whereas high-risk procedures, such as deep intra-articular procedures or those in immunocompromised patients, may benefit from more rigorous aseptic techniques.

While sterilisation methods are essential for infection control, their implementation faces real-world challenges. Financial constraints may limit access to sterile equipment, and proper training requires institutional support. Additionally, integrating effective disinfection measures into busy clinical workflows without causing delays remains a concern.

The main limitation of this review is the heterogeneity of methods and protocols between studies. Variations in sterilisation techniques, US-guided procedures, and infection control practices make it difficult to draw definitive conclusions. The lack of standardised criteria for assessing sterility further complicates the interpretation of results, as different measurement approaches lead to inconsistencies. In addition, many RCTs did not report skin or probe sterilisation methods, introducing bias and limiting the assessment of protocol effectiveness.

Future research should aim to address these limitations by focusing on controlled, large-scale studies that have standardised sterilisation protocols and assessment criteria. In addition, other studies should evaluate the feasibility of implementing standardised sterilisation protocols in different clinical settings, considering cost, training, and workflow integration. These studies should specify the sterilisation techniques used, including probe and skin disinfection and the use of sterile equipment. By developing standardised and universally accepted guidelines, future studies can provide more robust data on the effectiveness of different sterilisation methods in reducing infection and improving patient outcomes.

## 5. Conclusions

Interventional physiatry is an expanding branch of rehabilitation medicine, involving many physiatrists with growing enthusiasm and a large body of supporting literature. Although this study aimed to evaluate sterility during interventional procedures performed by physiatrists, the results were inconclusive. Taken together, findings of this scoping review showed that, to date, the scientific literature lacks a clear relationship between current sterilisation practices and the prevention of the microbial contamination of the probes or the patient’s skin. Variability in the disinfectants used, their application, and the specific protocols, followed by different healthcare professionals, contributed to the uncertainty of the results. This highlights the need for standardised guidelines and more rigorous studies to accurately determine the most effective disinfection practices.

To minimise the risk of infection during US-guided interventional procedures, physiatrists should maintain the highest level of sterility. The adherence to aseptic techniques, including the use of sterile gloves and a no-touch approach, significantly reduces the risk of contamination. Proper injection site preparation and minimising unnecessary needle repositioning further enhance infection prevention.

Although there was no clear support for a direct relationship between specific disinfection methods and sterility outcomes, the study highlights the importance of the adherence to hygiene protocols. This will not only improve patient safety but also ensure the optimal use and longevity of US equipment in clinical practice.

## Figures and Tables

**Table 1:** Overview of the most frequent diseases in the included studies.

Disease	Patients	Disease	Patients
Gleonohumeral OA	95	Shoulder pain	845
Acromion Clavicular OA	20	SASD bursitis	186
Elbow OA	80	Hemiplegic shoulder pain	111
Wrist	120	Tendinopathy	396
Trapezio-Metacarpal OA	121	Epicondylopathy	203
Hip OA	910	LBP	40
Knee OA	1151	Sacroiliac joint disease	190
Ankle OA	144	CTS	232
Diffuse inflammatory OA	408	Trigger Finger	36
AC	992	Knee effusion	64
Shoulder calcific tendinopathy	191	Bone fractures	30

AC, adhesive capsulitis; CTS, carpal tunnel syndrome; LBP, low back pain; OA, osteoarthritis; SASD, subacromial subdeltoid.

## Data Availability

The original contributions presented in this study are included in the article/Supplementary Materials. Further inquiries can be directed to the corresponding author.

## Supplementary Materials

The following supporting information can be downloaded at: https://www.mdpi.com/article/10.3390/diagnostics15070933/s1, Table S1: Overview of the included studies and a summary of their results.

## Abbreviations

The following abbreviations are used in this manuscript:

OA	osteoarthritis
RCT	randomised clinical trial
US	ultrasound
