# Supplementary material for: Impact of Adequate Disinfection Techniques for Ultrasound-Guided Injections in Musculoskeletal Rehabilitation: A Scoping Review
Source: Diagnostics (Basel). 2025 Apr 5;15(7):933. doi: 10.3390/diagnostics15070933 (PMC11989170; doi:10.3390/diagnostics15070933)
Supplement: Supplementary file 1 [file diagnostics-15-00933-s001.zip › diagnostics-3483623-supplementary.pdf]

Table 1. Overview of the included sudies and a summary of their results.

Author, year	Title	Sample	Intervention type	Skin disinfection method	US probe disinfection method	Side effects
Fu et al., 2024	Demonstrating the effectiveness of intra-articular prolotherapy combined with peri-articular perineural injection in knee osteoarthritis: a randomized controlled trial	60 patients with the diagnosis of knee OA	Random assignment to intra-articular prolotherapy group, Peri-articular perineural injection group or combined treatment group.	Not specified	Not reported	No adverse events
Hopewell et al., 2024	Anti-TNF (adalimumab) injection for the treatment of pain-predominant early-stage frozen shoulder: the AntiFreaze-Feasibility randomised controlled trial	156 patients with AC	US-guided intra-articular injection of: (1) adalimumab (160mg) or (2) placebo (saline (0.9% sodium chloride))	Not reported	Not reported	No adverse events
Wu et al., 2024	Efficacy of combined ultrasound-guided hydrodilatation with hyaluronic acid and physical therapy in patients with adhesive capsulitis: A randomised controlled trial	62 patients with AC	group A: ultrasound-guided hydrodilatation with hyaluronic acid + physical therapy ($N = 31$) and group B: physical therapy alone ($N = 31$)	Sterile technique	Not reported	No adverse events
Foula et al. 2023	Ultrasound-guided Shoulder Intraarticular Ozone Injection Versus Pulsed Radiofrequency Application for Shoulder Adhesive Capsulitis: A Randomized Controlled Trial	45 patients with primary AC.	3 groups: 1 intra-articular US-guided shoulder injection: steroid group, ozone group and pulsed radiofrequency group	Not reported	Not reported	Not reported
Moosmayer et al., 2023	Ultrasound guided lavage with corticosteroid injection versus sham lavage with and without corticosteroid injection for calcific tendinopathy of shoulder: randomized double blinded multi-arm study	220 adults with persistent calcific tendinopathy of the shoulder for at least three months	73 patients in the US-guided depot lavage plus subacromial injection of corticosteroid and lidocaine group; 74 patients in the US-guided sham lavage plus subacromial injection of corticosteroid and lidocaine group; 73 patients in the US-guided sham lavage plus subacromial injection of corticosteroid and lidocaine group.	Sterile technique	Not specified	No severe adverse events
Kirschner et al. 2022	Efficacy of ultrasound-guided glenohumeral joint injections of leukocyte-poor platelet-rich plasma versus hyaluronic acid in the treatment of glenohumeral osteoarthritis: a randomized, double-blind controlled trial.	70 patients with chronic glenohumeral OA	2 groups of 1 US guided intra-articular shoulder injection of 1) 6 mL HA, 2) 6 mL leukocyte-poor platelet-rich plasma (LP-PRP)	Not reported	Not reported	Side effect rates were 3.9% and 2.7% in the HA and LP-PRP groups, respectively. Weakness was the most common adverse event

Nouri et al. 2022	Comparison between the effects of ultrasound guided intra-articular injections of platelet-rich plasma (PRP), high molecular weight hyaluronic acid, and their combination in hip osteoarthritis: a randomized clinical trial.	105 patients with hip OA	3 US-guided hip joint injection Groups: 1) PRP, 2) HA 3) PRP + HA. Two injections with 2 weeks' interval in either group	"Sterile condition"	"Sterile condition"	1) Pain after injection was significantly different in the HA group, who experienced less pain after injection.<br>2) 17 patients from all three groups had complications such as warmth, stiffness, and heaviness with no significant difference.
Park et al., 2022	Usefulness of combined handheld ultrasound and fluoroscopy-guided injection in adhesive capsulitis of the shoulder: A prospective, randomized single blind-pilot study	39 patients with adhesive capsulitis of the shoulder	19 patients received combined hand-held US and fluoroscopy-guided corticosteroid injection.<br>20 patients received conventional US-guided corticosteroid injection.	Not reported	Not reported	No severe adverse events
Paskins et al. 2022	Clinical effectiveness of one ultrasound guided intra-articular corticosteroid and local anaesthetic injection in addition to advice and education for hip osteoarthritis<br>(HIT trial): single blind, parallel group, three arm, randomised controlled trial.	199 patients aged ≥40 years with hip OA	3 groups: A) Best Current Treatment (BCT) alone, B) BCT + US-guided intra-articular hip injection of 40 mg triamcinolone acetonide and 4 mL 1% lidocaine, C) BCT plus US-guided intra-articular hip injection of 5 mL 1% lidocaine.	Chlorhexidine 0.5% solution	The probe was covered with gel and a sterile sheath	In the BCT plus US triamcinolone-lidocaine arm 1) 6% of patients reported thinning or whitening at the injection site, 2) 6% had hot flushes, and 3) seven serious adverse events were recorded. One event was possibly related to study treatment: one participant with a bioprosthetic aortic valve died of subacute bacterial endocarditis.
Dório et al., 2021	Efficacy of platelet-rich plasma and plasma for symptomatic treatment of knee osteoarthritis: a double-blinded placebo-controlled randomized clinical trial.	62 patients with knee OA	3 groups: PRP (n= 20), plasma (n= 21) and saline (n= 21). Two US-guided knee injections were performed with a 2-week interval.	Not reported	Not reported	Participants in the PRP group had a significantly higher incidence of adverse events (65% compared to 25% in the plasma group and 33% in the placebo group). The most common adverse event reported was pain, typically mild to moderate in intensity, with

						an average duration of 2.1 days.
Emami Razavi et al., 2021	Short-term Efficacy of Ultrasonographic Guidance for Intra-articular Corticosteroid Injection in Hallux Rigidus: A Single-Blind Randomized Controlled Trial.	50 patients with Hallux Rigidus	Each patient received a single intra-articular injection of 40-mg methylprednisolone plus 1 mL lidocaine into the affected first metatarsophalangeal joint.	Povidone-iodine	Not reported	No severe adverse events
Hanson et al., 2021	Safety and Systemic Exposure of Triamcinolone Acetonide Following Ultrasound-Guided Intra-Articular Injection of Triamcinolone Extended-Release or Standard Triamcinolone Acetonide in Patients with Shoulder Osteoarthritis: An Open-Label, Randomized Study.	25 patients with shoulder OA	2 groups of single US guided intra-articular injections of: 1) extended-release triamcinolone 32 mg, 2) triamcinolone acetonide 40 mg	Not reported	Not reported	No serious adverse events. 7 mild adverse events (pain, muscle aches)
Hsieh et al., 2021	Comparison of the corticosteroid injection and hyaluronate in the treatment of chronic subacromial bursitis: A randomized controlled trial.	186 patients with chronic subacromial bursitis	3 groups of single SASD injections under US guidance: 1) 20mg triamcinolone; 2) 2.5mL HA; 3) 2.5mL normal saline.	Not reported	Not reported	Not reported
Jurgensmeier et al., 2021	Intra-articular Injections of the Hip and Knee with Triamcinolone vs Ketorolac: A Randomized Controlled Trial.	110 patients with moderate to severe radiographic primary OA (52 hips and 58 knees)	US-guided intra-articular injection of ketorolac or triamcinolone.	ChloraPrep ®	Sterile technique	Nausea, hypertension, and a flare-up of temporal arteritis were each reported once in those who received steroids. In the ketorolac group, one patient reported headache and nausea that resolved the day after injection, and a gastrointestinal bleeding was reported after a patient increased his warfarin dose about 2 months after ketorolac injection.
Paget et al., 2021	Effect of Platelet-Rich Plasma Injections vs Placebo on Ankle Symptoms and Function in Patients with Ankle Osteoarthritis.	100 patients with ankle OA	2 groups of 2 US guided intra-articular ankle injections of: 1) PRP (n= 48), 2) placebo (saline; n= 52)	Not reported	Not reported	1 serious adverse event (transient ischaemic attack) in the placebo group (not related to injection) and 13 non-severe adverse events (knee pain or leg muscle

						pain).
Roddy et al., 2021	Optimising outcomes of exercise and corticosteroid injection in patients with subacromial pain (impingement) syndrome: a factorial randomised trial	256 patients with subacromial pain syndrome	4 treatment groups: 1) US-guided corticosteroid injection + physiotherapist-led exercise, 2) US-guided corticosteroid injection + exercise booklet, 3) no US-guided corticosteroid injection + physiotherapist-led exercise, 4) no US-guided corticosteroid injection + exercise booklet.	Chlorhexidine 0.5% solution	Chlorhexidine 0.5% solution + sterile gel	Post-injection discomfort, local skin changes, presyncope, nausea or flushing in 13% of the US-guided injection groups and 13% of the unguided injection groups. Pain after exercise in 60% of the physiotherapist-led exercise groups and 59% of the leaflet groups. One serious adverse event: pyelonephritis in the US-guided injection and physiotherapist-led exercise groups.
Akbari et al., 2020	Ultrasound-guided versus blind subacromial corticosteroid and local anesthetic injection in the treatment of subacromial impingement syndrome: A randomized study of efficacy.	29 patients with subacromial impingement syndrome	14 patients received US-guided subacromial corticosteroid and anaesthetics injection and 15 patients received a blind subacromial corticosteroid and anaesthetics injection	10% povidone iodine solution	Not specified	No relevant adverse effects
Azadvari et al, 2020	Ultrasound-guided versus blind subacromial bursa corticosteroid injection for paraplegic spinal cord injury patients with rotator cuff tendinopathy: a randomized, single-blind clinical trial.	30 patients with paraplegic SCI	15 patients received a subacromial corticosteroid injection through anatomical landmarks and 15 patients received an injection guided by US.	Not specified	Not specified	Not reported
El Naggar et al., 2020	Effectiveness of radial extracorporeal shock-wave therapy versus ultrasound-guided low-dose intra-articular steroid injection in improving shoulder pain, function, and range of motion in diabetic patients with shoulder adhesive capsulitis.	103 diabetic patients with shoulder adhesive capsulitis (AC)	51 patients: single ultrasound-guided low-dose intra-articular steroid injection of 20 mg triamcinolone acetonide in the posterior short-axis of the shoulder.	Not reported	Not reported	No relevant adverse effects in the injections group.
Klontzas et al., 2020	The effect of injection volume on long-term outcomes of US-guided subacromial bursa injections	308 patients with periarticular shoulder pathology	US-guided corticosteroid injections into the SASD bursa	Cleaning and application of povidone-iodine	Sterile gloves and probe cover	Not reported
Kon et al.,	Autologous Protein Solution Injections for the	46 patients with	2 groups: 1 US-guided injection of APS (n= 31) or 1	Not reported	Not reported	No adverse events

2020	Treatment of Knee Osteoarthritis: 3-Year Results	Kellgren-Lawrence 2 or 3 knee OA	saline injection (n= 15)			
Louwerens et al., 2020	Comparing Ultrasound-Guided Needling Combined with a Subacromial Corticosteroid Injection Versus High-Energy Extracorporeal Shockwave Therapy for Calcific Tendinitis of the Rotator Cuff: A Randomized Controlled Trial.	82 patients with Calcific Tendinitis of the Rotator Cuff	41 patients received US-guided SASD injection	Sterile preparation and drapes	Not specified	2 patients developed a frozen shoulder. 5 patients returned to the outpatient clinic in the first 2 months with severe symptoms of subacromial bursitis.
Thu et al., 2020	Comparison of ultrasound-guided platelet-rich plasma injection and conventional physical therapy for management of adhesive capsulitis: a randomized trial	64 patients with adhesive capsulitis	US-guided injection of PRP into the shoulder joint (n=32)	Aseptic conditions	Not reported	No major adverse effects. In the PRP group, five patients had post-injection pain of non-severe intensity.
Villanova-López et al, 2020	Randomized, double-blind, controlled trial, phase III, to evaluate the use of platelet-rich plasma versus hyaluronic acid in hip coxarthrosis.	74 patients with hip OA not responding to conservative treatment.	US-guided hip infiltration via anterolateral approach	Not specified	Not specified	No relevant adverse effects
Yiannakopoulos et al., 2020	Ultrasound-guided versus palpation-guided corticosteroid injections for tendinosis of the long head of the biceps: A randomized comparative study.	44 patients with tendinosis of the long head of the biceps	22 patients: US-guided injection to the bicipital groove	Not specified	Not specified	No relevant adverse effects
Babaei-Ghazani, et al., 2019	A Randomized Control Trial of Comparing Ultrasound-Guided Ozone (O2-O3) vs Corticosteroid Injection in Patients with Shoulder Impingement.	30 patients with shoulder pain and clinical signs and symptoms of impingement	Single US-guided injection into the subacromial bursa	Antiseptic	Sterile barrier	No relevant adverse effects
Coory et al., 2019	Efficacy of suprascapular nerve block compared with subacromial injection: a randomized controlled trial in patients with rotator cuff tears	42 participants with symptomatic rotator cuff tears	26 patients received US-guided suprascapular nerve block. 26 patients received US-guided subacromial corticosteroid injection	Not reported	Not reported	Not reported
Darrieutort-Laffite et al., 2019	Are corticosteroid injections needed after needling and lavage of calcific tendinitis? Randomised, double-blind, non-inferiority trial.	132 patients with symptomatic shoulder calcification measuring more than	Patients received 1mL of saline or steroid in the subacromial bursa at the end of US-guided puncture and lavage	Not specified	Not specified	Mild vagal reactions occurring in 12 patients (9%)

		5mm.				
Lin et al., 2019	Effects of hypertonic dextrose injection in chronic supraspinatus tendinopathy of the shoulder: a randomized placebo-controlled trial.	31 patients with chronic supraspinatus tendinopathy and shoulder pain for more than six months.	16 patients received US-guided hypertonic dextrose prolotherapy injection; 15 patients received US-guided normal saline injection.	Not reported	Not reported	No adverse events
Roh et al., 2019	Comparison of Ultrasound-Guided Versus Landmark-Based Corticosteroid Injection for Carpal Tunnel Syndrome: A Prospective Randomized Trial	102 patients with CTS	2 groups: landmark-based injection and US-guided injection.	Skin was sterilized	Sterile cover	Symptoms of median nerve irritation 1, skin discolouration or subcutaneous fat atrophy 1, steroid flares 2
Wang et al., 2019	Ultrasound-Guided Standard vs Dual-Target Subacromial Corticosteroid Injections for Shoulder Impingement Syndrome: A Randomized Controlled Trial	60 patients with subacromial impingement syndrome	US-guided standard subacromial bursa; dual-target (subacromial bursa plus proximal biceps long-head tendon) injection	Not specified	Not specified	Not reported
Babaei-Ghazani et al., 2018	The effects of ultrasound-guided corticosteroid injection compared to oxygen–ozone (O2–O3) injection in patients with knee osteoarthritis: a randomized controlled trial.	62 patients with knee OA	31 patients received triamcinolone (1 cc) injected into the knee under US guidance 31 patients received 10 cc (15 µg/ml) oxygen-ozone (O2-O3) injected into the knee under US guidance	Sterilization of the skin	The US probe was covered with a sterile barrier and a sterile gel was used	No adverse effect
Huang et al., 2018	The long-term effects of hyaluronic acid on hemiplegic shoulder pain and injury in stroke patients	27 patients with Hemiplegic shoulder pain after stroke.	ultrasound-guided subacromial 0.9% sodium chloride (N=9) and HA injections (N=18),	Not specified	Not specified	No reports of harmful side effects
Jones et al., 2018	A randomized, controlled study to evaluate the efficacy of intra-articular, autologous adipose tissue injections for the treatment of mild-to-moderate knee osteoarthritis compared to hyaluronic acid: a study protocol.	54 patients with mild-to-moderate knee OA	A single intra-articular US-guided injection of autologous adipose tissue or a single intra-articular US-guided injection of HA (1:1 ratio).	Not specified	Not specified	Not reported
Malahias et al., 2018	Image-guided versus palpation-guided injections for the treatment of chronic lateral epicondylopathy: a randomized controlled clinical trial	44 patients with chronic epicondylopathy	2 groups of 22 people: Group A patients received three US-guided betamethasone injections; Group B received three "blind" injections, centred on the palpable point of maximum pain.	Sterile conditions	Sterilized surgical glove of the probe	Not reported
Malahias et	Platelet-Rich Plasma versus Corticosteroid	33 patients with	16 patients received 2 US-guided HA-PRP injections,	Sterilization of the	Coverage of the US	Not reported

al., 2018	Intra-Articular Injections for the Treatment of Trapezio-metacarpal Arthritis: A Prospective Randomized Controlled Clinical Trial.	Trapezio-metacarpal OA	while 17 patients received 2 US-guided intra-articular methylprednisolone and lidocaine injections at 2-week intervals.	skin	probe with a sterile pad, use of appropriate gel	
Rahimzadeh et al., 2018	The effects of injecting intra-articular platelet-rich plasma or prolotherapy on pain score and function in knee osteoarthritis.	42 patients with knee OA	42 patients (21 in each group): PRP therapy group received 7 mL of separated plasma US-guided intra-articular injection. Patients in the PRL group received 7 mL of 25% dextrose US-guided intra-articular injection.	Sterile conditions	Not reported	Not reported
Eker et al., 2017	The efficacy of intra-articular lidocaine administration in chronic knee pain due to osteoarthritis: A randomized, double-blind, controlled study	52 patients with knee OA	Group I (n= 26) received US-guided intra-articular injections of 7 mL of 0.5% lidocaine and group II (n= 26) received US-guided intra-articular injections of 7 mL of saline into the painful knee for a series of three injections spaced one week apart.	Not reported	Not reported	Not reported
Karrahmet et al., 2017	Comparing the effectiveness of ultrasound-guided versus blind steroid injection in the treatment of severe carpal tunnel syndrome.	40 hands of patients with severe CTS	19 hands received blind carpal tunnel steroid injection; 21 hands received US carpal tunnel steroid injection	Sterilization of the skin	Not specified	No adverse event
Kianmehr et al., 2017	A randomized blinded comparative study of clinical response to surface anatomy guided injection versus sonography guided injection of hyaluronic acid in patients with primary knee osteoarthritis.	61 patients with primary knee OA	31 patients received blind injection of HA into the knee; 30 patients received US guided injection of HA into the knee.	Antiseptic protocol	Antiseptic protocol	Not reported
Kon et al., 2017	Clinical Outcomes of Knee Osteoarthritis Treated with an Autologous Protein Solution Injection: A 1-Year Pilot Double-Blinded Randomized Controlled Trial.	46 patients with unilateral knee OA	APS group (n= 31) received a single US-guided injection of APS. The saline (control) group (n= 15) received a single US-guided saline injection.	Not reported	Not reported	No adverse events
Merolla et al., 2017	Arthroscopic Debridement Versus Platelet-Rich Plasma Injection: A Prospective, Randomized, Comparative Study of Chronic Lateral Epicondylitis with a Nearly 2-Year Follow-Up.	101 patients with chronic lateral epicondylitis	50 patients received arthroscopic release; 51 patients received US-guided PRP injections	Iodine solution	Not specified	No adverse event
Raeissadat et al., 2017	Comparing the accuracy and efficacy of ultrasound-guided versus blind injections of steroid in the glenohumeral joint in patients	41 patients diagnosed with shoulder AC.	Patients were randomised to receive intra-articular injections either blind (21) or under ultrasound guidance (20).	Not reported	Not reported	No adverse effects occurred.

	with shoulder adhesive capsulitis.					
Singla et al., 2017	Steroid vs. Platelet-Rich Plasma in Ultrasound-Guided Sacroiliac Joint Injection for Chronic Low Back Pain.	40 patients with sacroiliac joint pathology	20 patients received 1.5 mL methylprednisolone (40mg/mL) and 1.5 mL 2% lidocaine with 0.5 mL saline. 20 patients received 3 mL of leukocyte-free PRP with 0.5 mL of calcium chloride.	Not reported	Not reported	No major complications were observed
Wang et al. 2017	Short-term effect of ultrasound-guided low-molecular-weight hyaluronic acid injection on clinical outcomes and imaging changes in patients with rheumatoid arthritis of the ankle and foot joints. A randomized controlled pilot trial.	44 patients with RA having unilateral or bilateral painful ankle and foot involvement (N=75)	All patients were randomized to receive HA (N=40) or lidocaine (LI) (N=35) injections at 2-week intervals;	Aseptic technique	Sterilized plastic covering	Not reported
Ammitzbøll-Danielsen et al., 2016	Intramuscular versus ultrasound-guided intra tenosynovial glucocorticoid injection for tenosynovitis in patients with rheumatoid arthritis: a randomized, double-blind, controlled study.	50 patients with RA and tenosynovitis	25 patients received an intramuscular injection of betamethasone and a US-guided intra-tenosynovial injection of isotonic saline, and 25 patients received an intramuscular injection of saline and a US-guided intra-tenosynovial injection of betamethasone.	'No touch technique'	'No touch technique'	No serious adverse events were registered
Arendt-Nielsen et al., 2016	Intra-articular onabotulinumtoxinA in osteoarthritis knee pain: effect on human mechanistic pain biomarkers and clinical pain.	121 patients with knee OA	Single US-guided IA injection of onabotA (200 U)	Not reported	Not reported	No adverse events
Cho et al., 2016	Proper site of corticosteroid injection for the treatment of idiopathic frozen shoulder: Results from a randomized trial.	110 patients with AC	All participants were randomized to receive US-guided intra-articular, subacromial or combined injections.	Not specified	Not specified	No adverse event
Cole et al., 2016	Ultrasound-Guided Versus Blind Subacromial Corticosteroid Injections for Subacromial Impingement Syndrome: A Randomized, Double-Blind Clinical Trial.	56 shoulders with subacromial impingement syndrome	2 groups: 28 shoulders received a subacromial corticosteroid injection with US guidance, and 28 shoulders received a subacromial corticosteroid injection without US guidance.	Not reported	Non-sterile gel	Not reported
Dallari et al., 2016	Ultrasound-Guided Injection of Platelet-Rich Plasma and Hyaluronic Acid, Separately and in Combination, for Hip Osteoarthritis: A Randomized Controlled Study.	111 patients with Hip OA	All patients were randomised to 3 groups and received 3 weekly injections of either PRP (44 patients), PRP+HA (31 patients) or HA (36 patients).	Sterile condition	Sterile cover	No relevant side effects
Di Sante et al., 2016	Intra-articular hyaluronic acid vs platelet-rich plasma in the treatment of hip osteoarthritis.	43 patients with unilateral severe hip OA	22 patients received 3 US-guided intra-articular injections of PRP; 21 patients received 3 US-guided intra-articular injections of HA	Not specified	Not specified	No adverse event

Ellegaard et al., 2016	Exercise therapy after ultrasound-guided corticosteroid injections in patients with subacromial pain syndrome: a randomized controlled trial	99 patients with subacromial pain syndrome	All participants received two steroid injections into the painful shoulder with an interval of one week.	Not reported	Not reported	Not reported
Giordano, 2016	Comparison of Two Injection Techniques for Intra-articular Hip Injections	40 patients with clinical evidence of intra-articular hip pain	All participants received a single intra-articular corticosteroid injection into the hip using either an automated delivery system or a traditional syringe injection.	Chloraprep stick™	Chlorhexidine swab. Sterile Pre-Packaged US gel.	Not reported
Huang et al, 2016	The effects of hyaluronic acid on hemiplegic shoulder injury and pain in patients with subacute stroke: A randomized controlled pilot study.	26 subacute stroke patients with hemiplegic shoulder injury and pain	2 groups: the experimental group (n=16) received ultrasound-guided subacromial HA injections once a week for 3 weeks and conventional rehabilitation, while the control group (n=10) received 0.9% sodium chloride injections once a week for 3 weeks and conventional rehabilitation.	Not reported	Not reported	No adverse events
Karkucak et al., 2016	Education and Visual Information Improves Effectiveness of Ultrasound-Guided Local Injections on Shoulder Pain and Associated Anxiety Level: A Randomized Controlled Study.	151 patients with shoulder pain	Patients in group I (n=72) were given information about the US findings and were allowed to observe the procedure from the monitor, whereas patients in group II (n=79) received only the injection without any collaboration.	Sterile conditions	Sterile conditions	No side effect
Lee et al., 2016	Capsule-preserving hydro dilatation with corticosteroid versus corticosteroid injection alone in refractory adhesive capsulitis of shoulder: a randomized controlled trial	64 patients with AC	32 patients received US-guided corticosteroid and lidocaine injection; 32 patients received US-guided capsule hydro dilatation with corticosteroid, lidocaine, and normative saline.	Not reported	Not reported	Minor side effects (facial flushing, dizziness)
Namazi et al., 2016	Investigating the effect of intra-articular PRP injection on pain and function improvement in patients with distal radius fracture.	30 patients who had intra-articular distal radius fractures	15 cases received intra-articular autologous PRP	Not reported	Not reported	No adverse events
Soneji et al., 2016	Comparison of Fluoroscopy and Ultrasound Guidance for Sacroiliac Joint Injection in Patients with Chronic Low Back Pain.	40 with chronic LBP secondary to SIJ arthritis	US- or Fluoroscopic-guided unilateral SIJ injections	Asepsis	Asepsis	No significant adverse effects
Gutierrez et al., 2015	Short-term efficacy to conventional blind injection versus ultrasound-guided injection of local corticosteroids in tenosynovitis in patients with inflammatory chronic arthritis: A randomized comparative study.	114 patients with tenosynovitis in patients with inflammatory chronic arthritis	All participants were randomized to receive conventional blind injection (54 patients) or ultrasound-guided injection (60 patients) of corticosteroids	Not specified	Not specified	Not reported

Haghighat et al., 2015	Effectiveness of Blind & Ultrasound Guided Corticosteroid Injection in Impingement Syndrome.	40 patients with impingement syndrome	20 patients per group were allocated randomly to blind steroid injection group or ultrasound-guided steroid injection group	Sterile condition	Sterilization of the probe	Not reported
Liu et al., 2015	Ultrasound-guided hyaluronic acid injections for trigger finger: a double-blinded randomized controlled trial.	36 patients (39 affected digits) with diagnosis of trigger finger	All subjects were randomized into HA (12 digits) and steroids (17 digits) US-guided injection group	Not specified	Not specified	No adverse event
Prestgaard et al., 2015	Ultrasound-guided intra-articular and rotator interval corticosteroid injections in adhesive capsulitis of the shoulder: a double-blind, sham-controlled randomized study.	122 patients with AC	All patients were randomized (42 to intra-articular injection, 40 to combined intra-articular/interval injection, and 40 to sham injection).	Not reported	Not reported	2 patients reported severe chest or shoulder pain in the first few days after intra-articular injection, while one patient had mild pain. One patient in the combined injection group reported severe shoulder pain, 3 patients had mild discomfort and pain, and one patient reported elevated blood sugar. One patient in the sham injection group reported a rash and one patient reported nausea.
Dragoo et al. 2014	Platelet-Rich Plasma as a Treatment for Patellar Tendinopathy. A Double-Blind, Randomized Controlled Trial.	23 patients with patellar tendinopathy on examination and MRI	13 patients received US-guided dry needling injection; 10 patients received US-guided dry needling injection with leukocyte-rich PRP injection.	Not specified	Not specified	No adverse events
Jee et al. 2014	Ultrasound-Guided Versus Fluoroscopy-Guided Sacroiliac Joint Intra-Articular Injections in the Noninflammatory Sacroiliac Joint Dysfunction: A Prospective, Randomized, Single-Blinded Study.	110 patients with non-inflammatory sacroiliac OA	55 patients received intra-articular sacroiliac joint injections under fluoroscopic guidance; 55 patients received intra-articular sacroiliac joint injections under US guidance.	Not specified	Not specified	Not reported
Kim et al., 2014	Which method is more effective in treatment of calcific tendinitis in the shoulder? Prospective randomized comparison between ultrasound-guided needling and extracorporeal shock wave therapy.	54 patients diagnosed with unilateral painful shoulder calcific tendinitis	US needling or ESWT group	10% iodopovidone solution for 3 times and antiseptically draped	Not reported	No major side effects

Laver et al., 2014	Plasma rich in growth factors (PRGF) as a treatment for high ankle sprain in elite athletes: a randomized control trial.	16 elite athletes with anterior-inferior tibio-fibular ligaments tear	8 patients received US-guided PRP injections in the antero-inferior tibio-fibular ligaments + physiotherapy; 8 patients received physiotherapy only.	Not specified	Not specified	Not reported
Lee et al., 2014	Effectiveness of ultrasound-guided carpal tunnel injection using in-plane ulnar approach: a prospective, randomized, single-blinded study.	75 cases of 44 patients diagnosed with CTS	All patients received injection with 40 mg of triamcinolone mixed with 1 mL of 1% lidocaine into the carpal tunnel using an in-plane Ultrasound (US)-guided ulnar approach, out-plane US-guided approach, and blind injection.	Skin antisepsis	Not reported	Blind group: 5 nerve insult, 2 vascular insult, 8 skin lesion. Ulnar out of plane: 6 nerve insult, 1 skin lesion. Ulnar in plane: 1 nerve insult, 3 skin lesion.
Monfort et al., 2014	Comparative efficacy of intra-articular hyaluronic acid and corticoid injections in osteoarthritis of the first carpometacarpal joint: results of a 6-month single-masked randomized study.	88 patients diagnosed with thumb OA	48 patients received US-guided intra-articular treatment with HA; 40 patients received the same type of treatment but with betamethasone	Not specified	Not specified	Minor or moderate local pain after injection
Nam et al., 2014	Palpation versus ultrasound-guided corticosteroid injections and short-term effect in the distal radioulnar joint disorder: a randomized, prospective single-blinded study	60 patients with distal radioulnar joint disorder	Randomly assigned to US-guided or palpation-guided IA injection.	iodopovidone and alcohol, covered with a sterilization wrap. aseptic gloves	Not reported	No severe complications.
Battaglia et al., 2013	Efficacy of ultrasound-guided intra-articular injections of platelet-rich plasma versus hyaluronic acid for hip osteoarthritis.	100 patients with chronic unilateral symptomatic hip OA	Group A received PRP; group B received HA administered via intra-articular US-guided injections.	Injection was sterilely performed	Not reported	No major complications or adverse events occurred
De Witte et al., 2013	Calcific Tendinitis of the Rotator Cuff: A Randomized Controlled Trial of Ultrasound-Guided Needling and Lavage Versus Subacromial Corticosteroids.	48 patients with calcific tendinitis of the rotator cuff	23 patients received US-guided needling and irrigation (barbotage) combined with US-guided corticosteroid injection into the subacromial bursa. 25 patients received a US-guided corticosteroid injection in the bursa.	Not specified	Not specified	No adverse event
Hsieh et al., 2013	Is Ultrasound-Guided Injection More Effective in Chronic Subacromial Bursitis?	92 patients with chronic subacromial bursitis	46 patients US-guided injection of a mixture of 0.5 mL dexamethasone suspension and 3 mL lidocaine into the subacromial bursa. 46 patients palpation-guided injection of the same treatment	Not specified	Not specified	Not reported
Jang et al., 2013	Comparison of ultrasound (US)-guided intra-articular injections by in-plain and out-of-plain on medial portal of the knee.	126 knees with OA radiographically documented	US-guided in-plain, out-of-plain, and blind knee intra-articular injection via the mid-medial portal.	Iodopovidone and alcohol, covered with a sterilization	Not reported	No major complications occurred

				wrap. Aseptic gloves		
Kesikburun et al., 2013	Platelet-rich plasma injections in the treatment of chronic rotator cuff tendinopathy: a randomized controlled trial with 1-year follow-up.	40 patients with rotator cuff tendinopathy	Patients received a US-guided injection into the subacromial space containing either 5 mL of PRP prepared from autologous venous blood or 5 mL of saline.	Not reported	Not reported	No major complications occurred
Kim et al., 2013	Ultrasound Versus Palpation Guidance for Intra-articular Injections in Patients with Degenerative Osteoarthritis of the Elbow.	80 patients elbow OA	40 patients received US-guided intra-articular elbow injections of iohexol contrast; 40 patients received palpation-guided intra-articular elbow injections of iohexol contrast.	Povidone-iodine	Not specified	Not reported
Park et al., 2013a	Comparison of Ultrasound-Guided Intra-Articular Injections by Long Axis in Plane Approach on Three Different Sites of the Knee.	40 patients with knee OA	Five ml of a mixture of 1% lidocaine (1 ml), 20 mg triamcinolone (1 ml) and non-ionic contrast (3 ml) was injected into the knee through three different ways.	Povidone-iodine and alcohol	Not specified	No adverse event
Park et al., 2013b	Treatment effects of ultrasound-guided capsular distension with hyaluronic acid in adhesive capsulitis of the shoulder.	90 patients with shoulder AC	45 patients in group A were treated with 0.5% lidocaine plus triamcinolone 40mg IA injection and 45 patients in group B were treated with 0.5% lidocaine + HA 20mg and capsular distension. Injections were given every 2 weeks for 3 times.	Not specified	Not specified	No severe complications were observed
Rabago et al., 2013	Hypertonic dextrose and morrhuate sodium injections (prolotherapy) for lateral epicondylosis (tennis elbow): results of a single-blind, pilot-level, randomized controlled trial.	26 adults (32 elbows) with lateral epicondylosis	3-arm randomized to ultrasound-guided PrT with dextrose, PrT with dextrose-morrhuate or watchful waiting (Wait-and-see).	Not reported	Not reported	No major complications occurred
Rha et al., 2013	Comparison of the therapeutic effects of ultrasound-guided platelet-rich plasma injection and dry needling in rotator cuff disease: a randomized controlled trial.	39 patients with a not complete supraspinatus tendon lesion	Two dry needling procedures in the control group and two PRP injections in the experimental group were applied to the affected shoulder at four-week intervals using US-guide.	Sterile field	Sterile probe cover	No major complications occurred
Üstün et al., 2013	Ultrasound-Guided vs. Blind Steroid Injections in Carpal Tunnel Syndrome: A Single-Blind Randomized Prospective Study.	46 patients with CTS	Patients were randomised to receive 23 patients to US-guided injection of 40 mg methylprednisolone into the carpal tunnel; or 23 patients to blind injection of 40 mg methylprednisolone into the carpal tunnel.	Not specified	Not specified	No adverse events

Yoon et al., 2013	Optimal dose of intra-articular corticosteroids for adhesive capsulitis: a randomized, triple-blind, placebo-controlled trial.	53 with primary adhesive capsulitis in the freezing stage	US-guided intra-articular injections of 40 mg triamcinolone acetonide (high dose group, n= 20), 20 mg triamcinolone acetonide (low dose group, n= 20) or placebo (n= 13).	Sterile technique	Sterile technique	Flushing on days 1-5 after injection (3 participants in the high-dose group and 1 in the low-dose group) and dizziness due to vasovagal reactions during injection (1 participant in the placebo group and 1 in the low-dose group).
Di Sante et al., 2012	Ultrasound-guided aspiration and corticosteroid injection compared to horizontal therapy for treatment of knee osteoarthritis complicated with Baker's cyst: a randomized, controlled trial.	60 patients with a knee OA and diagnosis of Baker's cyst confirmed	Patients were randomized to either the US-guided BC aspiration and corticosteroid injection group (group A), the horizontal therapy group (group B) or the US-guided BC aspiration and corticosteroid injection plus horizontal therapy group (group C).	Aseptic conditions	Not specified	Not reported
Dogu et al., 2012	Blind or ultrasound-guided corticosteroid injections and short-term response in subacromial impingement syndrome: a randomized, double-blind, prospective study.	46 patients with subacromial impingement syndrome	23 US-guided and 23 blind corticosteroid injections	Alcohol	Sterile gel	No adverse event
Rah et al., 2012	Subacromial corticosteroid injection on post stroke hemiplegic shoulder pain: a randomized, triple-blind, placebo-controlled trial.	58 post-stroke hemiplegic shoulder pain	Participants were randomly assigned to receive ultrasound-guided subacromial injection with triamcinolone 40mg (treatment group, n=29), or lidocaine (placebo group, n=29).	Sterile technique	Not specified	Flushing on days 1 to 5 after injection (2 participants in treatment group) and dizziness due to vasovagal reaction during injection (1 participant in placebo group)
Sibbit et al., 2012	Does ultrasound guidance improve the outcomes of arthrocentesis and corticosteroid injection of the knee?	64 palpably effusive knees	(i) palpation-guided arthrocentesis using a conventional 20ml syringe (22 knees), (ii) US-guided arthrocentesis using a 25ml mechanical aspiration syringe with a reciprocating procedure device (RPD) (22 knees), or (iii) US-guided arthrocentesis using a 60ml automated aspiration syringe (20 knees).	Not specified	Not specified	No adverse event
Zufferey et al., 2012	A controlled trial of the benefits of ultrasound-guided steroid injection for shoulder pain.	70 patients with acute shoulder pain	Patients were randomized to receive either a standard subacromial infiltration of 7 mg of betamethasone or a US-guided injection	Not reported	Not reported	Four patients reported flushing and one diabetic patient a transient hyperglycaemia
Atchia et	Efficacy of a single ultrasound-guided	77 hip OA	Standard care (no injection); normal saline; non-	"Aseptic technique"	"Aseptic technique"	There was one case of

al., 2011	injection for the treatment of hip osteoarthritis.		animal stabilised HA (Durolane) or methylprednisolone acetate (Depomedrone).			femoral head collapse in a patient in the standard care group (who therefore did not receive an injection), four patients in the Durolane group experienced a significant flare-up of symptoms within days of the injection. of symptoms within a few days of injection. There was one confirmed case of post-arthroplasty infection (Durolane group).
Abdelshafi et al., 2011	Relief of chronic shoulder pain: a comparative study of three approaches.	50 patients with chronic shoulder pain	Group 1 (23 shoulders) received continuous ultrasound-guided suprascapular nerve block in addition to the rehabilitation programme. Group II (20 shoulders) received intra-articular steroid injections in addition to the rehabilitation programme. Group III (20 shoulders) received a rehabilitation programme only.	Not specified	Not specified	Not specified
Bum Park et al., 2011	Accuracy of blind versus ultrasound-guided suprapatellar bursal injection.	99 patients with knee OA	Fifty patients were assigned to the US-guided injection group and 49 to the blind injection group. After US-guided or blind injection of HA and contrast agent through the suprapatellar bursa into the knee joint	Not specified	Latex-free transducer cover with sterile coupling gel	Not specified
Hashiuchi et al., 2011	Accuracy of the biceps tendon sheath injection: ultrasound-guided or unguided injection? A randomized controlled trial	30 patients with reported anterior shoulder pain and a primary diagnosis of tenosynovitis or biceps tendinitis, or both.	Patients were randomised into US-guided and non-US-guided injection groups.	Not specified	Not specified	Not specified
Hong et al., 2011	Comparison of high- and low-dose corticosteroid in subacromial injection for periarticular shoulder disorder: a randomized, triple-blind, placebo-controlled trial.	79 people with periarticular shoulder disorders	Participants were randomly assigned to receive US-guided subacromial injection with triamcinolone acetonide, 40 or 20mg, or placebo.	Sterile technique	Not specified	Flushing on days 3 to 6 (1 participant in group 2) and dizziness due to vasovagal reaction during injection (1

						participant in group 3)
Sibbit et al., 2011a	A randomized controlled trial evaluating the cost-effectiveness of sonographic guidance for intra-articular injection of the osteoarthritic knee.	94 non effusive OA knees	Conventional palpation-guided anatomical landmark injection or US-guided injection augmented by a one-handed mechanical syringe	Not specified	Not specified	Not specified
Sibbit et al., 2011b	A randomized controlled trial of the cost-effectiveness of ultrasound-guided intraarticular injection of inflammatory arthritis.	Joints with inflammatory arthritis (n= 244)	Randomised to injection by conventional palpation-guided anatomical injection (120 joints) or US-guided injection augmented with a one-handed reciprocating mechanical syringe (124 joints).	Not reported	Not reported	Not reported
Cunnington et al., 2010	A randomized, double-blind, controlled study of ultrasound-guided corticosteroid injection into the joint of patients with inflammatory arthritis.	184 patients with inflammatory arthritis and an inflamed joint (shoulder, elbow, wrist, knee, or ankle)	US-guided or clinical examination-guided corticosteroid injections	"Skin disinfection and aseptic technique"	"Skin disinfection and aseptic technique"	Not specified
Sabeti-Aschraf et al., 2010	The infiltration of the AC joint performed by one specialist: ultrasound versus palpation a prospective randomized pilot study.	20 patients affected by OA of the acromion-clavicular joint	Patients were randomly assigned to the "US" or the "palpation" group to perform injection of the AC joint	"The area was cleaned of the contact gel, and then cleaned sterilely"	Not specified	No adverse event
Lee et al., 2009	Randomized controlled trial for efficacy of intra-articular injection for adhesive capsulitis: ultrasonography-guided versus blind technique.	43 patients with AC	Patients received a 20mg intra-articular injection of triamcinolone mixed with 1.5mL of 2% lidocaine and 4mL of normal saline in the first week, followed by five weekly injections of sodium hyaluronate using either a US-guided or blinded technique.	Not specified	Not specified	Not specified
Ucuncu et al., 2009	A comparison of the effectiveness of landmark-guided injections and ultrasonography guided injections for shoulder pain.	60 consecutive patients with shoulder pain due to soft tissue disorders	randomly assigned to receive triamcinolone (40 mg) either by landmark guided (LMG, n=30) or US-guided (n=30) injection.	Not reported	Sterile gel	Not reported
Luz et al., 2008	Ultrasound-guided intra-articular injections in the wrist in patients with rheumatoid arthritis: a double-blind, randomised controlled study.	60 patients with RA and wrist synovitis.	Randomised to receive intra-articular wrist injections by blind IA injection or US-guided IA injection with a solution of 1.0 ml 2% lidocaine, 1.5 ml triamcinolone hexacetonide, 0.5 ml non-ionic contrast agent and 0.5 ml air.	Not reported	Not reported	Not reported
Zeisig et al., 2008	Pain relief after intratendinous injections in patients with tennis elbow: results of a	32 patients (36 elbows), with a long	One US-guided injection with the sclerosing agent polidocanol (group 1) or the local anaesthetic	Not reported	Not reported	No major complications occurred

	randomized study.	duration tennis elbow	lidocaine plus epinephrine (group 2).			
Galiano et al., 2007	Ultrasound-guided versus computed tomography-controlled facet joint injections in the lumbar spine: a prospective randomized clinical trial.	40 adult patients with chronic low back pain	US-guided facet joint injections versus CT-controlled interventions	Sterile technique	Probe sterile wrapping and sterile ultrasound gel	A 54-year-old female patient developed fluid retention with oedema of the legs and arms. However, it is unclear whether these symptoms are due to a side effect of steroids.
Rutten et al., 2007	Injection of the subacromial-subdeltoid bursa: blind or ultrasound-guided?	20 patients with impingement syndrome of the shoulder	Blind or US-guided injection in the SASD bursa	70% ethanol and sterile gel	70% ethanol and sterile gel	No complications or side effects occurred.
Qvistgaard et al., 2005	Intra-articular treatment of hip osteoarthritis: a randomized trial of hyaluronic acid, corticosteroid, and isotonic saline.	101 patients with hip OA	Three US-guided, intra-articular injections were given at a 14-day interval.	Non-touch technique	Non-touch technique	No complications or side effects occurred.
Fredberg et al., 2004	Ultrasonography as a tool for diagnosis, guidance of local steroid injection and, together with pressure algometry, monitoring of the treatment of athletes with chronic jumper's knee and Achilles tendinitis: a randomized, double-blind, placebo-controlled study.	48 athletes with severe symptomatic patellar tendinitis (24) or Achilles tendinitis (24) for more than 6 months.	3 US-guided peritendinous injections of steroid or placebo	Not specified	Not specified	Reversible atrophy occurred in 20/48 patients
Raynauld et al., 2003	Safety and efficacy of long-term intraarticular steroid injections in osteoarthritis of the knee: a randomized, double-blind, placebo-controlled trial.	68 patients with knee OA	IA injections of triamcinolone acetonide 40 mg (34 patients) or saline (34 patients) into the study knee every 3 months for up to 2 years.	Not reported	Not reported	No complications or side effects occurred.

Abbreviations: AC, adhesive capsulitis; APS, autologous protein solution; CTS, carpal tunnel syndrome; ESWT, Extracorporeal shock wave therapy; HA, Hyaluronic Acid; OA, osteoarthritis; PRP, Platelet-rich plasma; PrT, prolotherapy; RA, rheumatoid arthritis; SASD, Subacromial subdeltoid; SCI, Spinal cord injury; US, Ultrasound.
